# Memory consolidation from a reinforcement learning perspective

**DOI:** 10.3389/fncom.2024.1538741

**Published:** 2025-01-08

**Authors:** Jong Won Lee, Min Whan Jung

**Affiliations:** ^1^Center for Synaptic Brain Dysfunctions, Institute for Basic Science, Daejeon, Republic of Korea; ^2^Department of Biological Sciences, Korea Advanced Institute of Science and Technology, Daejeon, Republic of Korea

**Keywords:** simulation-selection model, offline learning, value, dyna, imagination, CA3, CA1

## Abstract

Memory consolidation refers to the process of converting temporary memories into long-lasting ones. It is widely accepted that new experiences are initially stored in the hippocampus as rapid associative memories, which then undergo a consolidation process to establish more permanent traces in other regions of the brain. Over the past two decades, studies in humans and animals have demonstrated that the hippocampus is crucial not only for memory but also for imagination and future planning, with the CA3 region playing a pivotal role in generating novel activity patterns. Additionally, a growing body of evidence indicates the involvement of the hippocampus, especially the CA1 region, in valuation processes. Based on these findings, we propose that the CA3 region of the hippocampus generates diverse activity patterns, while the CA1 region evaluates and reinforces those patterns most likely to maximize rewards. This framework closely parallels Dyna, a reinforcement learning algorithm introduced by Sutton in 1991. In Dyna, an agent performs offline simulations to supplement trial-and-error value learning, greatly accelerating the learning process. We suggest that memory consolidation might be viewed as a process of deriving optimal strategies based on simulations derived from limited experiences, rather than merely strengthening incidental memories. From this perspective, memory consolidation functions as a form of offline reinforcement learning, aimed at enhancing adaptive decision-making.

## Introduction

Müller and Pilzecker (1900) introduced the term “consolidation”, proposing that after successful encoding, a physiological process known as “perseveration” stabilizes memory representations, gradually reducing their susceptibility to interference from new learning (Lechner et al., 1999). Despite over a century of research, the underlying reasons for consolidation and the transformations memories undergo during this process remain incompletely understood. According to the most influential theory, the standard systems consolidation theory, memories, initially encoded and temporarily stored in the hippocampus, are gradually reorganized and distributed across the neocortex. This process ensures that memories become less dependent on the hippocampus over time and are integrated into broader cortical networks for stable, long-term storage (Squire and Alvarez, 1995).

Although substantial evidence supports the systems consolidation theory, there are also findings that challenge its main premises. For instance, recent studies have observed impaired episodic memory without a temporal gradient following hippocampal damage in both humans and animals (Sekeres et al., 2018; Sutherland and Lehmann, 2011; Sutherland et al., 2010). Similarly, brain imaging studies have revealed hippocampal activation associated with the recollection of vivid, detailed, context-specific memories spanning the entire lifespan (Moscovitch et al., 2016; Moscovitch et al., 2005; Sekeres et al., 2018). These observations have prompted the development of alternative theories.

One such alternative is the multiple trace theory, which posits that memories are not merely transferred from the hippocampus to the neocortex but remain reliant on the hippocampus indefinitely for detailed episodic recollection. According to multiple trace theory, each retrieval of an episodic memory generates a new trace or representation within the hippocampus, reinforcing the memory and enhancing its accessibility over time (Nadel and Moscovitch, 1997). In contrast to systems consolidation theory, multiple trace theory suggests that while semantic memories may become independent of the hippocampus, episodic memories remain hippocampus-dependent for vivid, context-rich retrieval.

Another proposal, the trace transformation theory, emphasizes dynamic, bidirectional interactions between the hippocampus and neocortex. Rather than depicting memory consolidation as a simple handover of responsibility from the hippocampus to the neocortex, trace transformation theory envisions a lifelong process of hippocampal-neocortical collaboration. This theory emphasizes the coexistence and interaction of different forms of memory, suggesting that consolidation involves ongoing reorganization and expression of memories based on hippocampal-neocortical dynamics (Sekeres et al., 2018; Winocur and Moscovitch, 2011; Winocur et al., 2010; Winocur et al., 2007).

Despite extensive debate and research on these theories, one critical aspect has received less attention: the selective nature of memory consolidation. After encoding, not all memories share the same fate—some are forgotten, while others persist for a lifetime, often in a transformed or reconstructed form. Systems consolidation is widely understood to involve extracting general information from specific experiences, resulting in the formation of gists, schemas, and semantic knowledge (Cheng, 2017; McClelland et al., 1995; Moscovitch and Gilboa, 2024). However, indiscriminate generalization of individual experiences may be maladaptive. For survival, prioritizing memories of critical events—such as visiting a location associated with a significant reward or encountering a predator at a specific time and place—provides a clear advantage. Understanding the mechanisms that drive such selective memory consolidation remains a key challenge in memory research.

It has been proposed that the slow consolidation of memories serves an adaptive function by allowing endogenous processes triggered by an experience to influence memory strength (Gold and McGaugh, 1975). Research indicates that adrenal stress hormones, such as epinephrine and cortisol, released during emotional arousal, play a crucial role in modulating memory strength based on the significance of the experience, with the amygdala mediating the effects of these hormones on memory consolidation (Paré and Headley, 2023; Roozendaal et al., 2007; Mcgaugh and Roozendaal, 2002). These findings suggest that experiences with behavioral significance are more likely to be consolidated due to their activation of the emotional arousal system. However, the specificity issue remains unresolved, as emotional arousal modulates memory over a broad time scale. Various stimulant drugs that act on epinephrine and cortisol pathways enhance memory consolidation when administered within minutes or even hours after training (Mcgaugh, 2000; McIntyre et al., 2012). Nonetheless, it remains unclear whether and how emotional arousal selectively impacts memories of the numerous sensory experiences preceding the arousal. This highlights the need for further research to uncover the mechanisms that govern the selection and prioritization of memories for consolidation.

In this article, we discuss the memory selection issue from a different perspective. This perspective is based on two relatively recent findings: that the hippocampus is involved not only in remembering the past but also in imagining the future, and that it encodes robust value information. We begin by summarizing evidence supporting the role of the hippocampus in imagination and then review evidence that the hippocampus, particularly the CA1 region, encodes robust value information. Next, we introduce the simulation-selection model, which we propose as a framework to explain the functions of the CA3-CA1 neural network. Finally, we draw parallels between our model and the Dyna reinforcement learning algorithm (Sutton, 1991), suggesting that hippocampal processes underlying memory consolidation may be conceptualized as a form of offline reinforcement learning—a mechanism for reinforcing valuable future strategies by recombining past experiences through simulation.

### Hippocampus and imagination

The hippocampus, a critical brain structure traditionally recognized for its role in memory encoding and retrieval, is increasingly understood as essential for imagination and simulating future events. In humans, research has shown that the hippocampus is fundamental for generating detailed and coherent imagined scenarios. Patients with bilateral damage to the medial temporal lobes, which include the hippocampus, exhibit significant impairments in imagining hypothetical episodes (Hassabis et al., 2007). Furthermore, as a key component of the default mode network, the hippocampus is not only activated during the recall of autobiographical memories but also while envisioning future scenarios (Addis et al., 2007; Szpunar et al., 2007). These findings indicate the hippocampus is essential for synthesizing elements of memory into cohesive hypothetical episodes.

Animal studies complement and extend these findings by revealing the underlying neural mechanisms that allow the hippocampus to support imagination and predictive thought. Research on hippocampal replay, a phenomenon where patterns of neuronal activity representing past experiences are reactivated, has been particularly informative. In rats, hippocampal place cells go through rapid sequential discharges during periods of quiet rest and sleep, mirroring the order of activity observed during active navigation (Lee and Wilson, 2002; Foster and Wilson, 2006; Diba and Buzsáki, 2007). Initially, these replays were thought to support the recall and consolidation of prior navigation experiences. However, subsequent studies revealed that hippocampal replays also involve novel recombinations of previously learned trajectories (Gupta et al., 2010). Further research demonstrated that the hippocampus engages in preplay, where neuronal sequences representing paths in a novel environment are activated before the animal encounters them (Dragoi and Tonegawa, 2011). These findings suggest that the hippocampus constructs forward-looking models of the world, enabling prediction and preparation for future scenarios. Thus, findings from human and animal studies converge on the idea that the hippocampus is not merely a repository for memories but a flexible, predictive system capable of constructing mental representations of the past, present, and future.

### Value representation in the hippocampus

Early efforts to identify value-related neural activity primarily focused on brain regions outside the hippocampus, such as the parietal cortex, frontal cortex, and basal ganglia (O’doherty, 2004; Lee et al., 2012a; Glimcher, 2014). However, an early human imaging study detected value-related BOLD signals in the hippocampus alongside well-established value-related regions like the orbitofrontal cortex and striatum (Tanaka et al., 2004). In rats, value-related neuronal activity was identified in the CA1 region of the hippocampus (Lee et al., 2012b). The strength and characteristics of these CA1 value signals were comparable to those observed in traditional value-related regions such as the orbitofrontal cortex and striatum (Shin et al., 2021). Moreover, CA1 value signals temporally overlapped with choice and reward signals, indicating that CA1 integrates the necessary information for computing reward prediction errors and updating reward values (Lee et al., 2012b).

Additional studies have corroborated these findings across species. Human imaging studies further showed value-related BOLD signals in the hippocampus (Bornstein and Daw, 2013; Dombrovski et al., 2020), and physiological recordings in monkeys demonstrated value-dependent neuronal activity in this region (Knudsen and Wallis, 2021). In mice, calcium imaging studies revealed robust value signals in both dorsal and ventral CA1, with population activity in dorsal CA1 neurons increasing as a function of value (Biane et al., 2023; Yun et al., 2023). Collectively, these findings establish that value-related hippocampal neural processes are conserved across species, including rats, mice, monkeys, and humans.

Comparisons along the hippocampal transverse axis revealed that CA1 exhibits significantly stronger value signals than CA3 or the subiculum, its main input and output structures (Lee et al., 2012b; Lee et al., 2017). This observation aligns with evidence that CA1 neurons, unlike CA3, remap their place fields in response to changes in reward locations (Dupret et al., 2010). Furthermore, chemogenetic inactivation of CA1, but not CA3, impaired value learning without affecting value-dependent action selection, indicating CA1’s critical role in valuation (Jeong et al., 2018). These findings suggest that value processing sets CA1 apart from other hippocampal subregions, establishing it as a key region for integrating valuation with other hippocampal mnemonic processes.

### Simulation-selection model

**Figure 1:**
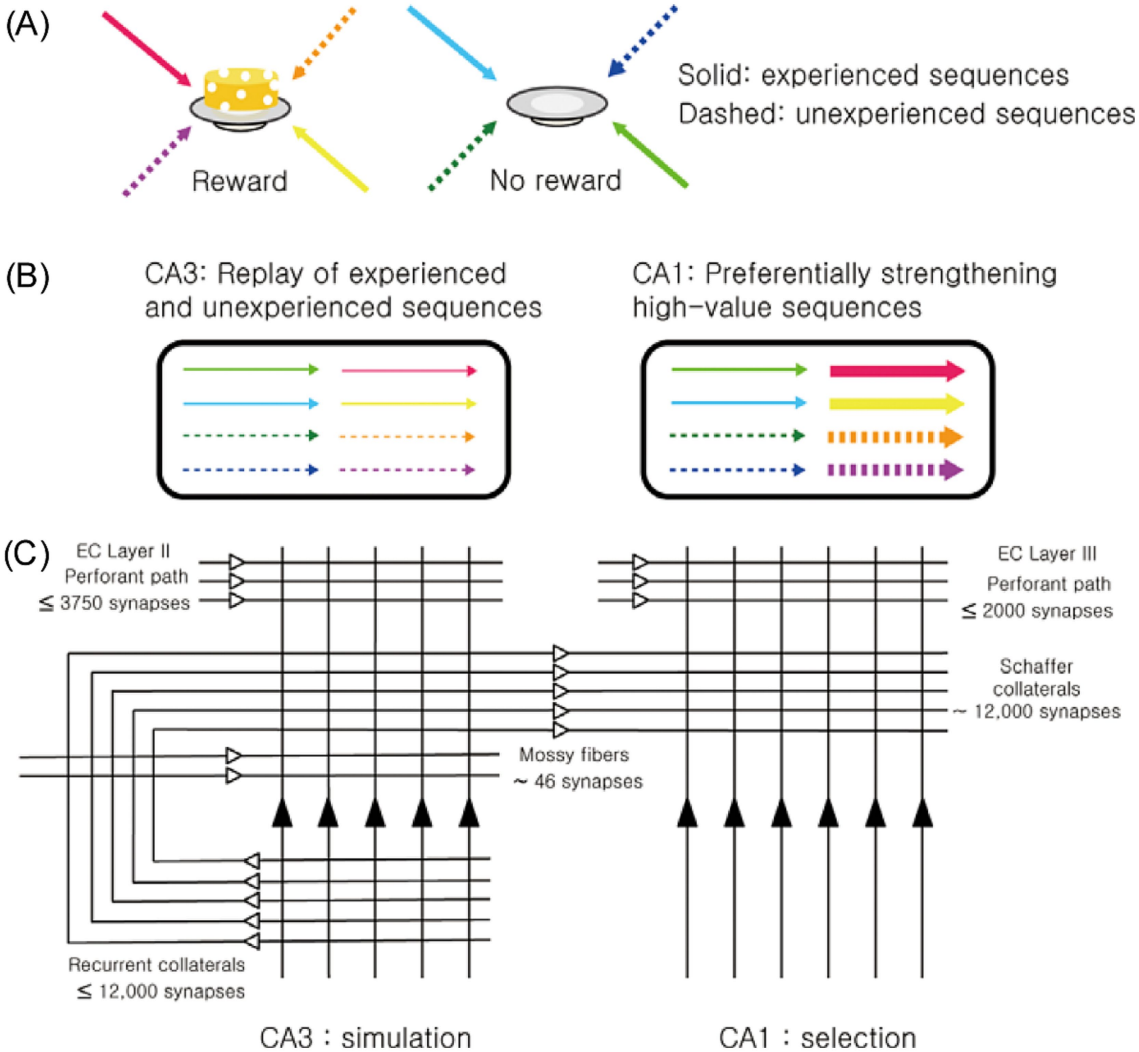
Overview of the simulation-selection model. **(A)** Navigation sequences to two locations—one where a reward was obtained (high-value sequence) and one where it was not (low-value sequence)—are represented using different colors. Solid arrows indicate experienced sequences, while dashed arrows represent unexperienced (novel) sequences. **(B)** CA3 generates both experienced and novel (unexperienced) navigation sequences, independent of their value. Among these, CA1 selectively reinforces high-value sequences, whether experienced or novel. **(C)** The schematic diagram illustrates the basic circuit organization of CA3 and CA1. The numbers denote the average number of synapses for each projection pathway in a single CA3 or CA1 pyramidal neuron (Amaral et al., 1990). The extensive but individually weak recurrent collaterals in CA3 enable the generation of both remembered (experienced) and novel (unexperienced) sequences. In contrast, CA1, which lacks recurrent collateral projections but conveys strong value signals, selectively reinforces high-value sequences. Figure adapted from Jung et al., 2018, licensed under CC-BY 4.0.

Building on discoveries indicating the hippocampus’s role in imagination and the CA1 region’s unique function in value processing, we propose a new framework for the CA3-CA1 neural network: the simulation-selection model (Jung et al., 2018). The model’s core concept is straightforward—CA3 acts as a simulator, generating diverse activity patterns, while CA1 functions as a selector, prioritizing and reinforcing patterns associated with high value (Figure 1). This selective reinforcement ensures that neural representations of high-value events and actions are strengthened, making them more likely to influence future decisions in similar contexts.

Anatomical and physiological evidence strongly supports CA3’s role in generating diverse activity patterns. A key anatomical distinction between CA3 and CA1 is the presence of extensive recurrent collateral projections in CA3. In rats, each CA3 pyramidal neuron receives approximately 12,000 Schaffer collateral synapses from other CA3 neurons, which comprise 75% of its excitatory inputs (Amaral et al., 1990). Because CA3 neurons are heavily interconnected, the activity of some neurons often triggers the activation of others (self-excitation), especially when inhibitory neuronal activity is low, such as during slow-wave sleep and quiet rest (Ranck, 1973). During these states, the hippocampus shows slow, irregular rhythmic activity interspersed with occasional sharp-wave ripples (SWRs)—synchronized neuronal discharges accompanied by 140–200 Hz oscillations (Buzsáki et al., 1992; Buzsáki and Vanderwolf, 1983; O'keefe, 1976). CA3 serves as the primary initiator of SWRs, during which the majority of hippocampal replays are observed (Buzsáki, 2015).

Several features of CA3’s architecture and dynamics facilitate the generation of novel activity patterns during SWRs (Jung et al., 2018). The CA3 network is characterized by many weak, recurrent synapses rather than a few strong ones (Debanne et al., 1999; Miles and Wong, 1986), making it more effective for generating variable sequences compared to networks dominated by fewer strong connections. Also, recurrent collateral synapses in CA3 support symmetric, rather than asymmetric, spike timing-dependent plasticity within a relatively broad time window (~150 ms) (Mishra et al., 2016). This promotes extensive associations among CA3 neurons with overlapping place fields, regardless of navigation trajectory. These features are consistent with the view that CA3 operates as a simulator, capable of generating activity patterns related to both previously experienced and unexperienced events.

In contrast, CA1 lacks the strong recurrent projections found in CA3, possessing only weak, short, longitudinally directed connections (Yang et al., 2014). Consequently, CA1 does not independently generate SWR-associated replays but instead processes activity sequences received from CA3. What distinguishes CA1 is its robust encoding of value, enabling it to process CA3-generated activity patterns differently based on their associated values. Although the value dependence of CA1 activity during SWRs remains incompletely characterized, existing findings strongly support this hypothesis. For example, CA1 place cells with firing fields near rewarding locations are preferentially reactivated during SWRs, whereas CA3 place cells do not exhibit such reward dependence (Dupret et al., 2010). Furthermore, CA1 replay preferentially encodes trajectories leading to reward locations (Foster and Wilson, 2006; Gupta et al., 2010; Ólafsdóttir et al., 2015; Pfeiffer and Foster, 2013). Reward also enhances the rate and fidelity of awake replays in CA1 (Ambrose et al., 2016; Bhattarai et al., 2020), which facilitates the consolidation of memories associated with these replays (Yang et al., 2024). In humans, rewards have been shown to enhance the imagination of episodic future events (Bulganin and Wittmann, 2015) and to preferentially reactivate high-reward contexts during post-learning rest, improving memory retention (Gruber et al., 2016; Sterpenich et al., 2021).

Collectively, these findings support the core premise of the simulation-selection model: CA3 generates diverse activity patterns, while CA1 selectively reinforces those associated with high value. The functional outcome of this interplay is the prioritization of high-value activity patterns, strengthening their neural representations and enhancing the likelihood of optimal future decision-making. The simulation-selection model generates numerous testable predictions. For instance, it predicts that CA1 replays will be more value-dependent than CA3 replays and that blocking CA3–CA1 synaptic plasticity during exploration will diminish the value dependence of CA1 replays (Jung et al., 2018). Additionally, the model suggests differential effects of CA3 and CA1 modulation on the diversity and value dependence of hippocampal replays, although the serial organization of the CA3–CA1 circuit poses challenges for interpreting such results. While further research is needed to test these predictions and address unresolved questions, such as differentiating the processing of positive and negative values, current findings align with this model and suggest its potential relevance in adaptive decision-making.

### Offline reinforcement learning

Reinforcement learning is a branch of artificial intelligence focused on discovering optimal action strategies in dynamic and uncertain environments. A central concept in reinforcement learning is the value function, which estimates the expected cumulative reward an agent can achieve from a specific state (or state-action pair) by following a particular strategy (policy). Agents use value functions to select actions and continuously update these functions based on the outcomes of their decisions. Through this iterative process, agents approximate true value functions and adapt their choices accordingly (Sutton and Barto, 1998). However, this trial-and-error approach can be highly inefficient, often requiring a vast number of trials to converge to accurate value estimates. This inefficiency becomes especially problematic in scenarios where achieving a goal involves long action sequences or where the environment changes rapidly.

To address these challenges, the Dyna algorithm, introduced by Richard Sutton in 1991, provides an integrated approach that significantly enhances learning efficiency. Dyna combines direct interactions with the environment and simulated experiences to accelerate the reinforcement learning process. During direct interaction, the agent collects data by exploring the environment and updating its value functions and policies based on observed outcomes. In parallel, the agent builds an internal model of the environment, capturing relationships between states, actions, and rewards. Using this model, the agent performs offline simulations to generate additional experiences, which are then used to further refine value functions and policies (Sutton and Barto, 1998; Sutton, 1991). This dual approach leverages the strengths of both model-free and model-based learning. The use of direct interaction ensures robustness and adaptability to environmental variations, while simulations enable faster learning and more efficient exploration of the state space by allowing the agent to explore hypothetical scenarios without real-world trials.

The similarity between the simulation-selection model and the Dyna algorithm is striking. The Dyna algorithm overcomes the inefficiencies of pure trial-and-error learning by combining real-world experiences with simulated planning, enabling agents to learn and adapt efficiently in complex and dynamic environments. Similarly, the simulation-selection model allows agents to navigate their environments more efficiently by complementing actual experiences with the simulation and evaluation of diverse scenarios, such as spatial trajectories, during idle states. Both frameworks accelerate value learning by leveraging simulations derived from limited experiences. In this analogy, the key components of the Dyna algorithm map seamlessly onto the simulation-selection model: the recurrent network architecture of CA3 supports the generation of simulated trajectories, functioning analogously to model-based planning, while the strong value signals in CA1 facilitate the selection and reinforcement of these simulations, akin to refining value functions and policies in Dyna.

At the outset, we noted that the ultimate goal of memory consolidation remains unclear. The simulation-selection theory offers a novel perspective, suggesting that memory consolidation is not merely the transformation or reorganization of temporary experiences stored in the hippocampus into long-term memory. Instead, it may function as a process of finding optimal strategies for navigating an environment through simulation, using limited experiences as a foundation. In this process, high-value behavioral strategies are selectively reinforced, facilitating better decision-making in the future. From this viewpoint, memory consolidation can be understood as a form of offline reinforcement learning. This idea also aligns with the constructive episodic simulation hypothesis, which posits that individuals flexibly extract and recombine elements of past experiences to simulate potential future scenarios (Schacter and Addis, 2007). Such flexibility allows past information to be effectively repurposed for simulating alternative future possibilities, reducing the reliance on actual trial-and-error behavior.

## Conclusion

Recent studies increasingly highlight the hippocampus’s role in predictive coding, emphasizing its capacity to prepare the brain for future scenarios (Barron et al., 2020; Buckner, 2010; Pezzulo et al., 2014). Theoretical advancements further support this view, with reinforcement learning frameworks emerging as powerful tools to explain hippocampal functions (Ambrogioni and Ólafsdóttir, 2023; Gershman and Daw, 2017; Stachenfeld et al., 2017; Tessereau et al., 2021). Integrating the simulation-selection model into this framework, we propose that hippocampal neural processes underlying memory consolidation might be understood as a form of offline reinforcement learning. From this perspective, memory consolidation is not a passive process of fortifying memories based on initial encoding strength or arousal level but an active process of selecting and reinforcing valuable options for the future by recombining past experiences through imagination (Cowan et al., 2021; Jung, 2023; Jung et al., 2018).

This perspective does not negate other proposed roles of memory consolidation, such as schema formation, emotional memory modulation, or semantic abstraction. Rather, it broadens current thinking on memory consolidation by positioning reinforcement learning as a valuable theoretical framework for understanding hippocampal processes. It emphasizes the hippocampus’s dual role in retaining past experiences and actively transforming them into actionable strategies for navigating future challenges. By bridging neural, behavioral, and computational frameworks, this approach provides new insights into the mechanisms and biological functions of hippocampal memory consolidation.

## Data Availability

The original contributions presented in the study are included in the article/supplementary material, further inquiries can be directed to the corresponding authors.
